# Safety and Efficacy of Probiotic Supplementation in Reducing the Incidence of Infections and Modulating Inflammation in the Elderly with Feeding Tubes: A Pilot, Double-Blind, Placebo-Controlled Study, “IntegPRO”

**DOI:** 10.3390/nu13020391

**Published:** 2021-01-27

**Authors:** Paolo Orlandoni, Nikolina Jukic Peladic, Angela Amoruso, Marco Pane, Mirko Di Rosa, Jennifer Vedruccio, Franco Santini

**Affiliations:** 1 Clinical Nutrition Unit, National Institute of Health and Science on Aging, IRCCS INRCA Ancona, Via della Montagnola 81, 60127 Ancona, Italy; rackog@yahoo.it; 2 Probiotical Research Srl, Via E.Mattei 3, 28100 Novara, Italy; a.amoruso@mofinalce.it (A.A.); m.pane@probiotical.com (M.P.); 3 Unit of Geriatric Pharmacoepidemiology and Biostatistics, National Institute of Health and Science on Aging, IRCCS INRCA Ancona, Via Santa Margherita 5, 60124 Ancona, Italy; m.dirosa@inrca.it; 4 Residenza Conero Santo Stefano, SS 16 Via Flaminia 293 326/A, 60020 Ancona, Italy; j.vedrux@gmail.com; 5 Ex Medical Direction, Errekappa Euroterapici S.p.A., Via Ciro Menotti 1/A, 20129 Milan, Italy; satirofancin@gmail.it

**Keywords:** probiotics, enteral nutrition, geriatric patients, inflammation

## Abstract

A double-blind, placebo-controlled study was performed in a sample of geriatric patients treated with home enteral nutrition (HEN) to analyze the efficacy of a probiotic supplement Proxian^®^, which contains *Lactiplantibacillus plantarum* LP01 (LMG P-21021), *Lentilactobacillus buchneri* Lb26 (DSM 16341), *Bifidobacterium animalis* subsp. *lactis* BS01 (LMG P-21384), and is enriched with zinc (Zn) and selenium (Se), in reducing the incidence of infections and modulating inflammation. Thirty-two subjects were enrolled (mean age 79.7 ± 10.3 years), 16 in the intervention group, 16 controls. They received Proxian^®^ or placebo for 60 days. Patients were assessed at baseline (t_0_) and 60 (t_1_) and 90 (t_2_) days after the beginning. Infections were detected by information regarding their clinical manifestations and the incidence of antibiotic therapy. Levels of C-reactive protein (CRP) were measured to study inflammation. Information on bowel function, nutritional status and testimonials regarding the feasibility of administration of the product were collected. Differences between the two groups in number of infections (25% intervention group vs. 44% controls), antibiotic therapies (12% vs. 37%) and modulation of CRP levels (median CRP moved from 0.95 mg/L (t_0_)_,_ to 0.6 (t_1_) and 0.7 (t_2_) in intervention group vs. 0.7 mg/L, 0.5 and 0.7 in controls) did not reach statistical significance. No significant changes in bowel function and nutritional status were found. Caregivers’ adherence was 100%. Results of this “IntegPRO” study showed that Proxian^®^ is potentially safe, easy to administer and promising for further studies but it appears not to change the incidence of infections or modulate inflammation in elderly treated with HEN. The utility of Proxian^®^ in reducing the incidence of infections and modulating inflammation in these subjects needs to be investigated by a larger multi-center clinical trial, and by using additional analyses on inflammatory markers and markers of infections.

## 1. Introduction

Elderly patients treated with home enteral nutrition (HEN) are particularly prone to developing infections. The origin of their increased susceptibility may be attributed to alterations of the immune system caused by underlying chronic pathologies and by immunosenescence, which are both characterized by an increased inflammatory state (inflammaging) [1,2]. The inflammatory state may be modulated by a number of factors, and an increasingly important role has been attributed to the regulation of intestinal microbiota. It is documented in different populations and relative to different pathologies that microbiota may be regulated with the use of probiotics containing different bacterial strains that reduce and modulate the inflammatory state [3,4,5,6,7,8,9]. Probiotics are generally considered safe. Some concerns have been raised about the safety of probiotics in immunocompromised and critically ill patients but the evidence is still inconclusive in that field [10,11]. In elderly subjects, a great variety of bacteria with pro-inflammatory effects have been found, while the administration of *Lactiplantibacillus plantarum*, both when it was administered alone or in combination with other strains, was associated with a reduction of overall infections in critically ill patients treated with artificial nutrition [12,13,14,15,16,17,18]. This preliminary, double-blind, placebo-controlled study was performed in a sample of geriatric patients recruited from the HEN Center at the Clinical Nutrition Unit of Scientific Institute for Research, Hospitalization and Healthcare (IRCCS), National Institute on Health and Science of Aging INRCA (Ancona, Italy). The aim was to test the efficacy of a specific probiotic supplement, namely, Proxian^®^, in reducing the incidence of infections and modulating inflammation in those subjects and to assess its safety and feasibility of administration. Proxian^®^ contains *Lactiplantibacillus plantarum* LP01 (LMG P-21021)—formerly named *Lactobacillus plantarum*, *Lentilactobacillus buchneri* Lb26 (DSM 16341)—formerly named *Lactobacillus buchneri, Bifidobacterium animalis* subsp. *lactis* BS01 (LMG P-21384), and is enriched with zinc (zinc gluconate) and selenium (sodium selenite). 

## 2. Materials and Methods

### 2.1. Study Design

**Figure 1:**
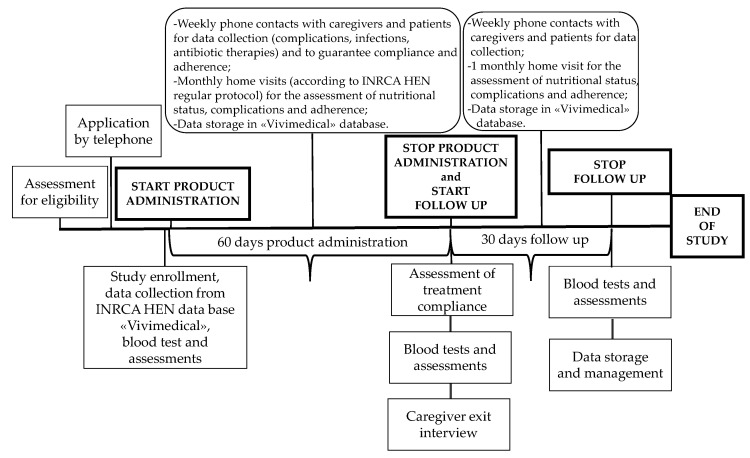
The timeline of the IntegPRO study procedure.

IntegPRO was a 90-day, placebo-controlled, double blind randomized controlled trial (RCT) that involved 60 days of administration of a specific product, Proxian^®^, a supplement of probiotics enriched with zinc and selenium, and 30 days of post administration follow-up, which was performed in order to test its ability to modulate the inflammation and modify the incidence of infections in geriatric patients treated with HEN. This trial was registered retrospectively on 9 July 2020 in the ISRCTN registry (ISRCTN75739497). Participants were recruited from the HEN Center of the Clinical Nutrition Unit of National Institute on Health and Science of Aging IRCCS INRCA (Ancona, Italy) in the period from 2 July 2018 to 15 June 2019. Participants were randomized to receive either Proxian^®^ or placebo. Participants in both groups completed assessments at baseline before the program commencement, and after 60 and 90 days with the primary and secondary outcomes measured at each step (see Figure 1). The study protocol of the IntegPRO study was approved on 20 September 2018 by the ethics committee of INRCA (approval n. 337/DGEN) in compliance with national rules and regulations in Italy. Reporting of findings was done in accordance with the Consolidated Standards of Reporting Trials (CONSORT) 2010 guidelines and their extension to non-pharmacologic treatments.

### 2.2. Participants

#### 2.2.1. Inclusion Criteria

Participants who were treated with HEN by the INRCA service for at least 30 days before enrollment were eligible. Details of the INRCA HEN service have been described in the literature [19]. Eligibility criteria included only participants that were older than 65 years and were not treated with antibiotics in the 30-day period prior to the study. Patients capable of understanding and communicating received the informed consent form and approved their own participation. Patients with severe cognitive impairment had to have the legal administrator authorized to provide written informed consent and approve the patient’s participation. Caregivers also had to agree to participate and to respect the protocol regarding the times and methods of administration and assessments.

#### 2.2.2. Exclusion Criteria

Patients with diagnoses of pancreatitis [20], hypersensitivity to any ingredient in the supplement, which was assessed in the patient or caregiver interview with regard to any previous reactions and side effects to the ingredients contained in the products that would be administered, as well as subjects who participated in other clinical studies or used probiotics in the 30-day period prior to the study were excluded.

### 2.3. Sample Recruitment

Patients and caregivers of patients who satisfied the inclusion criteria were contacted by phone and invited to participate after all the details about the study protocol were explained by researchers. The recruitment was a continuous process that started on 3 October 2018 and finished on 15 June 2019.

### 2.4. Interventions

The intervention group was administered with a dose of Proxian^®^ for 60 days via enteral tube. One single dose sachet of Proxian^®^/day dissolved in water contained the following doses of probiotic microorganisms: *B. lactis* BS01 ≥ 1 billion live cells/dose microencapsulated, *L. plantarum* LP01 ≥ 1 billion live cells/dose microencapsulated, tyndallized *L. buchneri* Lb26 (20 mg), zinc gluconate (1.5 mg) and sodium selenite (27.5 mcg). The daily doses of Proxian^®^ and duration of administration were defined based on the characteristics of the product. Bacteria of Proxian^®^ (Probiotical S.p.a., Novara, Italy) are microencapsulated with gastroresistant material so that their survival in gastric juice is almost totally complete, because the coating material does not dissolve in the acidic environment of the stomach. The evaluation of survival of the strains following the microncapsulation was tested in vitro, internally, on all microencapsulated strains by gastric pH survival analysis and flow cytometry analysis. In light of this evidence, it was possible to reliably estimate that at least 90% of the microencapsulated cells are able to survive passage through the stomach and the duodenum [21,22]. As such, the daily dose of Proxian^®^ is one stick/day for at least two months. Proxian^®^ is enriched with selenium and zinc. The control group was administered one single dose sachet of placebo/day dissolved in water, for 60 days. Placebo was composed of 100% flavorless maltodextrin. Both probiotic supplements and placebo were produce and provided by Probiotical S.p.A, Novara, Italy. Products were administered by caregivers one hour after dosing with the enteral formula. Caregivers received 60 sachets of product at enrollment. They received detailed information on how to administer products and were provided with supporting written information specifically designed to ensure correct storage of the products, their correct administration and regular communication between patients and/or caregivers and researchers on all issues possibly attributable to the administration of the products. In particular, patients and caregivers were invited to inform the Clinical Nutrition Unit about any gastrointestinal complication described in the written material. The hospital physician and nursing home health staff were responsible for assessing whether complications were possibly related to the administration of the probiotic and if the administration should be interrupted for patient safety. Patients and caregivers were free to withdraw from the study at any time. In order to ensure compliance and adherence, patients and caregivers were contacted by phone on a weekly basis. Treatment compliance was evaluated by counting the remaining supplements and placebos, and subtracting from the number of supplements and placebo (60) provided to caregivers.

### 2.5. Assessments and Outcomes

Once the participants and/or caregivers had signed written informed consent, the following baseline (t_0_) data were collected from the database at the Clinical Nutrition Unit: basic demographic information (age, sex, living conditions), data on general clinical conditions, concomitant diseases (primary pathology, comorbidities), nutritional status and nutritional therapy (body mass index (BMI), enteral formula administered, and route of administration), and information on infections and antibiotic therapy for the 30 days prior to enrollment. Blood tests were performed at the hospital’s laboratory. CRP values were detected to analyze inflammatory status [23,24]. According to the hospital laboratory where all the tests were performed, the inflammation status is defined by CRP values greater than or equal to 0.8 mg/dl. All data were attributed to the personal data of patients, which are stored and managed within a dedicated database “Vivimedical” that was created and is managed in compliance with the EU data protection policy.

#### Primary and Secondary Outcomes

Relative to the primary outcomes, the onset of infections was observed by detection of clinical manifestations of infections (prevalence) and antibiotic therapy (prevalence) during the product administration (60 days) and the follow-up period (30 days after the last administration). The inflammation was assessed by CRP values in the intervention group and in controls following the administration of products (Proxian^®^ and placebo) and lab analyses were performed at baseline, during the product administration (60 days) and the follow-up period (30 days after the last administration). Despite the fact that three blood tests had been scheduled by protocol, only two tests were performed (60 and 90 days from the start) due to the refusal of some caregivers to subject patients to blood tests too frequently. For secondary outcomes, the assessment of the motility was performed by collecting the evidence on bowel function and its variations as reported by caregivers at the time of phone contact at t_1_ and t_2_. Nutritional status was analyzed by assessing the BMI at each step (baseline, 60 and 90 days after enrollment). The feasibility of administering the probiotic supplement to the HEN population was tested by specific questionnaires administered to caregivers while adherence was stimulated by regular phone contacts. 

### 2.6. Sample Size 

The sample size was estimated based on the CRP variation from baseline to follow-up for cases and assuming a null effect on controls. According to the literature an effect size (Cohen’s f) of 0.23 was hypothesized [24]. Assuming 80% power, 0.05 alpha error, an allocation ratio between cases and control of 1 and 3 measurements, it was estimated that the overall sample size needed to capture such an effect size was 32 subjects (16 in the intervention group and 16 in the placebo group) in repeated measures analysis of variance model. Considering a 20% drop-out rate, overall, 40 subjects (20 cases and 20 controls) should be enrolled. The GPower (Heinrich-Heine-Universität Düsseldorf, Düsseldorf, Germany) software was used to estimate this sample size.

### 2.7. Randomization, Allocation Concealment and Blinding

A simple randomization process was performed. A computer-generated randomization technique based on a single sequence of random assignments was used. A list of computer-generated random numbers was used and subjects were assigned a number based on their order of inclusion in the study. The sequence was saved to a password-protected spreadsheet. Subjects were randomly assigned to one of the 2 study groups: the intervention group that was given Proxian^®^ and the control group, which was given placebo. The randomization was managed by the trial dieticians, and the randomization schedule and coding of group allocations were not accessible to research assistants conducting the assessments or to the biostatisticians. Subjects who were enrolled but withdrawn from the study after receiving at least one dose of the study product were replaced. For replacement, the simple randomization process was performed. Substitutes were randomly and blindly allocated between the two braces and the enrollment proceeded until there were 32 subjects—the sample size that had been pre-established by sample size estimation—who completed the 60-day administration of study product or placebo and the 30-day follow-up period. The equal group sizes (16 subjects in each group) were reached by chance.

Of 43 subjects enrolled, 11 subjects were lost. To prevent selection bias, the packaging of the two products, Proxian^®^ and the placebo, was identical, and products were marked by a code. The treatments were both tasteless and identical in appearance (white microgranules). Caregivers and/or patients and the researcher were blinded to prevent observation bias. Statistical analyses were conducted by an external statistician (SC), who was blind to group allocation prior to analysis.

### 2.8. Statistical Analysis

Per protocol analyses were adopted. Descriptive statistics were used to describe the patients’ characteristics at baseline, compare the frequency of clinical manifestations of infections and antibiotic therapy after the administration of the product, and compare CRP values in the two groups. Continuous variables were expressed as mean values ± standard deviation. Categorical variables were expressed as absolute values and relative frequencies. Independent samples’ t-tests and Fisher test analyses were used to compare both baseline characteristics and outcomes between groups. The primary efficacy analysis was based on between-group differences in prevalence of the primary outcome measures (infections and antibiotic therapy) following 60-day administration of the products, (Proxian^®^) and controls (placebo). Statistically significant differences between groups were evaluated with the χ^2^ test. The secondary efficacy analysis was based on between-group differences in inflammation, measured by the prevalence of subjects with CRP ≥ 0.8 mg/L among subjects who did not register clinical manifestations of infections throughout the study. The secondary outcome progression was evaluated by comparing the measure at three time-point: baseline, 60 days and 90 days. Whilst acknowledging the increased potential for type 1 errors, given that reported comparisons for all primary and secondary outcomes were pre-planned comparisons that were determined a priori and documented in the trial protocol, we did not make adjustments for multiple comparisons. All tests for significance used a two-sided *p*-value of 0.05. Data were analyzed with STATA 15.1 (StataCorp, College Station, TX, USA).

## 3. Results

**Figure 2:**
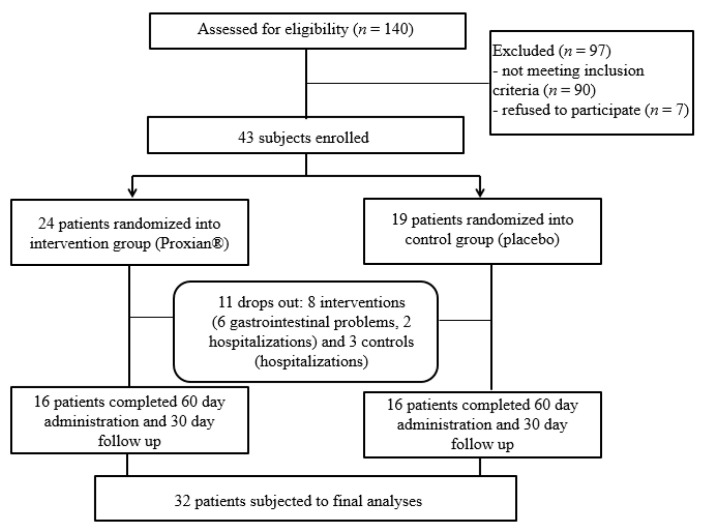
Flow chart of the IntegPRO study.

One hundred and forty patients treated with HEN at the INRCA HEN Center at the start of the IntegPRO study were assessed for eligibility and 50 patients (36%) were identified as eligible (see Figure 2). As many as 88 subjects (63%) had cognitive impairment but did not have a support administrator who could decide on their participation in the study and give a written consent while two patients (1%) did not meet the inclusion criteria because they were already taking probiotics. Seven patients (5%) were excluded because their caregivers did not want to participate, as they feared for the security and safety of the very old, frail and bedridden patients they were caring for.

Gastrointestinal problems (diarrhea) and the fear of caregivers that gastrointestinal problems could be due to probiotic supplementation were the reasons for refusing to proceed with administration in six subjects (all from the intervention group); five subjects were lost due to hospitalization for complications of underlying diseases (two from the intervention group and three controls). The final study population was composed of 32 subjects who completed all phases of the trial and were subject to final analyses. This was the minimum sample size required for statistical analyses.

Baseline characteristics of the two groups are shown in Table 1. The mean age of subjects enrolled was 79.7 ± 10.3 years. Subjects were residing prevalently in nursing homes (NH) (63%). No statistically significant differences were found at baseline between the two groups relative to their overall clinical conditions.

The most frequent comorbid conditions were different progressive neurological disorders (more than 30%), cardiovascular diseases (19%), type 2 diabetes (13%) and respiratory diseases (9%) without significant differences between the two groups. High prevalence of polypharmacy, which was assumed as more than five drugs, was registered in both groups [25]. There was a large proportion of underweight patients (BMI < 21); 63% in the intervention group vs. 50% in the placebo group. Inflammation was identified in 75% of subjects from the intervention group (CRP values ≥ 0.8 mg/L) vs. 50% in the placebo group. In the intervention group, there were far fewer enteral formulas with fiber administered compared to the placebo arm and this was the only statistically significant difference (7% vs. 56%, respectively; *p* < 0.05).

One hundred percent treatment adherence was reached during the study period. Forty-five percent of caregivers declared that the administration of product was very simple, 55% of them found it simple. Compliance with the protocol regarding blood sampling was more problematic. Some caregivers, despite having been fully informed about the study protocol and having accepted it, were reluctant to subject the patient to frequent blood tests. In response to this problem, the protocol underwent some changes, and instead of the three blood tests initially foreseen after the enrolment at 30, 60 and 90 days, two blood tests were performed (at 60 and 90 days).

After the 60-day period of administration of Proxian^®^ and placebo, the frequencies of clinical manifestations of infections and antibiotic therapies were compared (Table 2).

**Figure 3:**
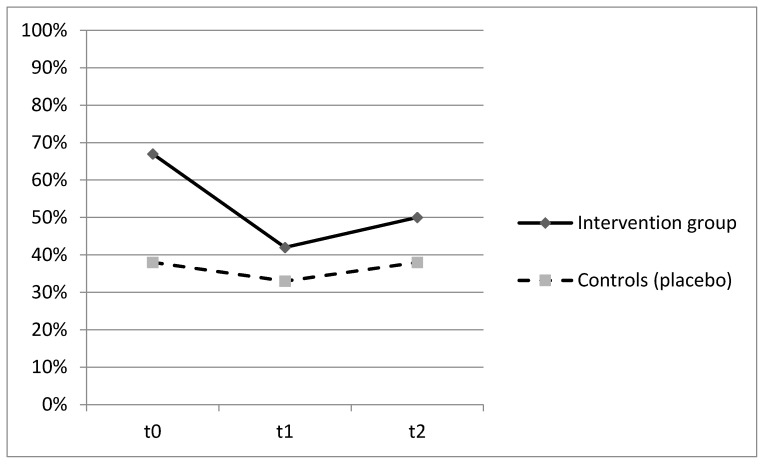
Prevalence of subjects with CRP ≥ 0.8 mg/L at t_0_ (baseline), t_1_ (60 days after enrollment) and t_2_ (90 days after enrollment) among subjects who did not register clinical manifestations of infection throughout the study—intervention group (Proxian^®^) and controls (placebo).

Patients who were administered with Proxian^®^ had fewer clinical manifestations of infections in the 60-day period but the differences between the two groups did not reach statistical significance. The main infections were urinary tract infections (45%), followed by gastrointestinal and enteric infections (27%), and respiratory tract infections (18%). No significant differences between the two groups were found with reference to single infection types. A lower incidence of bacterial infections in the intervention group was also confirmed by the lower number of antibiotic therapies in subjects who were given the probiotic supplement although, once again, the observed differences were not statistically significant. No adverse effects were registered. The ability of Proxian^®^ to modulate the inflammation was further tested by the analysis of changes in CRP levels in a subgroup of subjects who did not register clinical manifestations of infections during the study period. The results are shown in Figure 3.

The prevalence of subjects with CRP values ≥ 0.8 mL/L decreased in both groups with no statistically significant differences (Figure 3). At baseline, the percentage of subjects with elevated values of CRP was higher in the intervention group (67%) but it decreased following the administration of Proxian^®^ (42%). In the control group, it moved from 38% at baseline to 32% following the administration of placebo. The median value of CRP in the intervention group at baseline was 0.95 mg/L (min 0.2; max 4.9), which decreased to 0.6 mg/L following the administration of Proxian^®^. While it increased after the last administration, its levels were still below the cut-off value for elevated inflammation (0.7 mg/L; min 0.0; max 10.8). In contrast, the median CRP value of subjects in the placebo group was less than 0.8 mg/L at baseline (min 0.0; max 2.3); although there was some variation during the study period, the same level was seen at the end of follow-up (at t_2_ median 0.7 mg/L; min 0.2; max 16.8).

In the analysis of secondary outcomes, no significant changes in bowel function and nutritional status before and after the administration of Proxian^®^ were found, either within the group or between groups. The weight moved from 50.0 ± 11.0 kg at t_0_ to 54.0 ± 9.3 kg at t_1_ in the intervention group and from 52.8 ± 11.2 to 52.4 ± 10.2 kg in controls. The change in weight was not statistically significant either for the intervention group or the control group (*p* > 0.05 for both groups). Half of the subjects in both groups suffered from constipation before and after the administration of the products and at the end of the administration period, more than 80 percent of the subjects from both groups were periodically being treated with laxatives. The adherence of both groups was excellent relative to administration of products. 

## 4. Discussion

This double-blind, placebo-controlled pilot study, IntegPRO, was performed to test the safety of a probiotic supplement containing *L. plantarum*, *L. buchneri B. lactis* and enriched with zinc and selenium, and its ability to modulate inflammation and reduce the incidence of infections in geriatric patients treated with HEN. The feasibility of the administration and its impact on motility and nutritional status were also analyzed as secondary objectives. The IntegPRO study has some shorcomings that must be highlighted. Firstly, it was performed in a very limited number of subjects and secondly, a probiotic supplement was administered for the minimum period required by the manufacturer, that is, only 60 days. This likely prevented the ability to perform an analysis with greater statistical power. These weaknesses limit the generalizability of the results. Furthermore, to detect whether the probiotic supplementation is able to modulate inflammation, we assessed variations in CRP. This marker is frequently used as an indicator of inflammation because it is very cost effective to measure and it is easy to use in daily practice. However, CRP is not the only marker for inflammation, and the most appropriate way to detect inflammation would be to measure the secretion of proinflammatory cytokines. Thus the utility of Proxian^®^ in reducing the incidence of infections and modulating inflammation in subjects treated with HEN should also be investigated by using markers of inflammation other than CRP together with markers of infections. For statistical analyses, per protocol analyses was adopted while intent to treat analyses is a recommended method to avoid bias.

Numerous studies have been performed to detect the potential of probiotics for the treatment of various pathologies in different populations. As reported by Whelan K. and Myers CE., out of 53 trials that they analyzed in their systematic review, as many as 50 showed that probiotics have either no effect or a positive effect on infections and mortality [26]. Different strains were positively associated with reduction of infections and mortality. In particular, Temptè et al. and Bleichen et al. found that the administration of *S. boulardii* was beneficial for reduction of the episodes of diarrhea in critically ill adults. In 2002, Rayes et al. analyzed the efficacy of living *Lactobacillus plantarum* administered with enteral nutrition in a group of 95 fifty-year-old patients and found out that the rate of postoperative infections was significantly reduced after its administration [27]. Falcao et al. analyzed the efficacy of Lactobacillus johnsonii in 23 critically ill adults with brain injury and found that both the infections and the length of hospital stay were reduced in the intervention group [28]. In 2007, Spindler-Vesel et al. analyzed the impact of synbiotic containing *Pediococcus pentosaceus*, *Lactococcus raffinolactis*, *Lactobacillus paracasei* subsp *paracasei* and *Lactobacillus plantarum* in a group of 113 patients (mean age 41.0 ± 18.9) and found that the incidence of infections was significantly reduced in the intervention group [29]. The administration of *Lactiplantibacillus plantarum* was found to be associated with a reduction of overall infections in critically ill patients treated with artificial nutrition by Kecskés et al., McNaught et al. and Klarin et al. [16,17,18]. On the contrary, in 2008, Besselink et al. reported on the results of their study performed in 298 patients (mean age 60 years) with severe acute pancreatitis, and advised against administration of multispecies probiotic preparations containing *Lactobacillus acidophilus, Lactobacillus casei, Lactobacillus salivarius, Lactococcus lactis, Bifidobacterium bifidum*, and *Bifidobacterium lactis* given that higher mortality rates and bowel ischemia were registered among those patients [20]. Some concerns have also been raised about the safety of probiotics in immunocompromised and critically ill patients. The efficacy of probiotic interventions in those subjects has not been proven, and what is more, there might be a risk that in immunocompromised patients, probiotics might be immunogenic and/or pathogenic [10,11]. In older people, changes in the composition of microbiota following the assumption of probiotics and the efficacy of probiotics on symptoms of major gastrointestinal diseases such as constipation, diarrhea secondary to the assumption of antibiotics and *Clostridium difficile* are the most studied issues. Studies cited by Rondanelli et al. in their systematic review on the effectiveness of probiotic use in older patients, as well as those analyzed and cited by Salazar et al., suggest that probiotics may bring changes in the composition of the microbiota in those subjects, and be beneficial, mainly to reinforce the immune system and reduce the incidence of winter infections, or to treat undernutrition, and improve gastrointestinal motility [6,12]. Despite the large number of studies on probiotics, the quality of the scientific evidence is still insufficient. Studies used very different products, from fermented milk to products that contained very different bacterial strains, they were commonly performed on quite small samples and in very different populations, and the duration of the administration period differed consistently. The results of these studies are difficult to compare with each other and with the results of our study. Given that it was performed on a small sample of subjects, the IntegPRO study does not help to overcome those limitations. However it adds some new information on the safety and feasibility of the administration of a specific product, Proxian^®^ in elderly patients treated with HEN, and it draws the trajectories for future studies on its effectiveness in modulating inflammation and reducing the incidence of infections. Proxian^®^ is a probiotic supplement containing *L. plantarum*, *L. buchneri B. lactis*, and enriched with zinc and selenium. *Lactobacillus Buchneri* Lb26 internalizes Se, which has a potent antioxidant activity mediated through its ability to increase activity of the glutatione peroxidase enzymes. *Bifidobacterium lactis* BS01 internalizes Zn, which is an important element for growth and it is involved in numerous aspects of cellular metabolism. It plays a role in immune function protein and DNA synthesis and cell division. Different inorganic and organic forms of Zn and Se are currently used in numerous food supplements whereas probiotics enriched with these two minerals represent a very valid alternative to inorganic forms of such minerals, which are poorly absorbed through the gut mucosa and have a relatively low systemic effect [30]. The administration of Proxian in geriatric patients treated with HEN was simple and safe. In their review, Whelan and Myers list a number of case reports reporting adverse effects associated with the use of probiotics but underline that none of clinical trials reported them [24]. Although in our study some patients stopped the study due to gastrointestinal symptoms, those complications primarily occurred in patients residing at the same nursing home during an outbreak of viral gastroenteritis, and cannot be considered as confirmed adverse events associated with the use of the probiotic supplement, which was found to be safe. On the contrary, no statistically significant differences were found with reference to the incidence of infections and antibiotic therapy between the intervention group and controls (infections were 25% in the intervention group vs. 44% in the placebo; anthibiotic therapy 12% vs. 37%; *p* > 0.05), and the modulation of CRP in the intervention group did not differ significantly from that recorded by the control group. However, the results and suggestions emerging from this study deserve to be further developed within a larger study, on larger samples. The results of the IntegPRO study could be of benefit for setting up the correct methodological approach for a new study. In preparing for larger clinical trials, the difficulties encountered in enrolling patients must be considered. In the first place, the figure with legal representative, which is not yet widespread in Italy, is indispensable for patients with cognitive impairment, as geriatric patients often are. Secondly, the composition of the sample is often compromised to the disadvantage of the male population, as the female population lives longer. Therefore, more representative and sufficiently large samples can only be reached by performing multi-centric trials. Great attention must be paid to ensure patients’ safety and security and to reassure caregivers. Their adherence to intervention was excellent but too frequent blood test must be avoided. Some additional markers should be used to measure the inflammation (IL-6 and D-Dimer) and intention to treat analyses should be adopted. An additional analysis on circulating biomarkers of gut barrier function (zonulin, intestinal fatty acid binding protein I-FABP), and diamine oxidase (DAO) would also be useful. Finally, given that the two-month period of probiotic treatment represents the minimum treatment period indicated by the manufacturer, extending the analysis period could be considered.

In conclusion, the present study provides the first information regarding the safety and ease of administration of a specific probiotic, Proxian^®^, in a sample of elderly tube-fed patients. The IntegPRO study highlights the difficulties encountered in carrying out the study in elderly, frail subjects, and makes suggestions for a larger study that can better investigate the potential of Proxian^®^ to affect the modulation of inflammation and reduce the incidence of infections, which were not been demonstrated in this study.

## Figures and Tables

**Table 1:** Baseline characteristics of participants in the intervention group (Proxian^®^) and controls.

	Total (*n* = 32)	Intervention (*n* = 16)	Placebo (*n* = 16)	*p*
Age, Mean ± SD ^ (range) *	79.7 ± 10.3 (min 66; max 97)	80.0 ± 10.2 (min 67; max 93)	79.3 ± 10.0 (min 66; max 97)	0.853
Gender, *n* (%) **	F 24 (75%), M 8 (25%)	F 13 (81%), M 3 (19%)	F 11 (69%), M 5 (31%)	0.343
Living, *n* (%) **	Home 13 (41)%, NH 19 (59)%	Home 6 (38%), NH 10 (62%)	Home 7 (44%), NH 9 (56%)	0.500
Dementia, *n* (%) **	19 (59%)	11 (69%)	8 (50%)	0.236
Comorbidity, *n* (%) **	21 (66%)	12 (75%)	9 (56%)	0.229
BMI ^^, Mean ± SD ^ *	19.6 ± 5.1	19.5 ± 5.4	19.7 ± 5.2	0.898
Pressure ulcers, *n* (%) **	5 (16%)	4 (25%)	1 (6%)	0.166
Polypharmacy, *n* (%) **	21 (67%)	9 (57%)	12 (78%)	0.694
CRP ^^^ ≥ 0.8 mg/L, *n* (%) **	17 (53%)	9 (56%)	8 (50%)	0.500

* Student’s *t* test; ** Fisher’s exact test for comparison of intervention to control group. ^ Standard deviation; ^^ Body Mass Index; ^^^ C- reactive protein.

**Table 2:** Prevalence of infections and antibiotic therapy following the administration of products in the intervention group (Proxian^®^) and controls (placebo).

	Total (*n* = 32)	Intervention (*n* = 16)	Placebo (*n* = 16)	*p*
Clinical manifestations of infections, *n* (%) **	11(34%)	4(25%)	7(44%)	0.229
Antibiotic therapy, *n* (%) **	8(25%)	2(12%)	6(37%)	0.110

** Fisher’s exact test for comparison of intervention to control group.

## Data Availability

The data presented in this study are available on request from the corresponding author. The data are not publicly available due to privacy restrictions.

## Registration

This trial was registered retrospectively on 9 July 2020 in the ISRCTN registry (ISRCTN75739497).
